# A panel of DNA methylation markers reveals extensive methylation in histologically benign prostate biopsy cores from cancer patients

**DOI:** 10.1186/s40364-014-0025-9

**Published:** 2014-12-12

**Authors:** Igor Brikun, Deborah Nusskern, Daniel Gillen, Amy Lynn, Daniel Murtagh, John Feczko, William G Nelson, Diha Freije

**Affiliations:** Euclid Diagnostics LLC, Crown Point, Indiana USA; Department of Statistics, University of California Irvine, Irvine, California USA; Consultants in Laboratory Medicine, Toledo, Ohio USA; Promedica Genitourinary Surgeons, Toledo, Ohio USA; Pathology Consultants, Michigan City, Indiana USA; Sidney Kimmel Comprehensive Cancer Center, Johns Hopkins School of Medicine, Baltimore, Maryland USA

**Keywords:** DNA methylation, Prostate cancer, Repeat biopsies, High grade prostatic intraepithelial lesions, Atypical small acinar proliferation, Early cancer diagnostics

## Abstract

**Background:**

Men with a negative first prostate biopsy will undergo one or more additional biopsies if they remain at high suspicion of prostate cancer. To date, there are no diagnostic tests capable of identifying patients at risk for a positive diagnosis with the predictive power needed to eliminate unnecessary repeat biopsies. Efforts to develop clinical tests using the epigenetic signature of cores recovered from first biopsies have been limited to a few markers and lack the sensitivity and specificity needed for widespread clinical adoption.

**Methods:**

We developed methylation-specific quantitative polymerase chain reaction assays for a panel of 24 markers that are preferentially methylated in prostate cancer. We modified the bisulfite conversion conditions to allow the integration of the methylation information from multiple markers. We determined the methylation status of the 24 markers in 213 prostate biopsy cores from 104 patients, 37 prostate cancer patients and 67 controls. We performed logistic regression on combinations of markers as well as the entire panel of 24 markers to identify the best candidates for a diagnostic test.

**Results:**

The marker panel differentiated between cancer cores and benign cores from non-cancer patients with 100% sensitivity and 97% specificity. Furthermore, the panel detected significant methylation in benign cores from prostate cancer patients that was not present in controls. Using methylation of 5 out of 24 to define a cancer case, the analysis of a single benign biopsy core identified 62% of prostate cancer patients undergoing repeat biopsies. ROC curve analysis showed that markers commonly methylated in benign cores from cancer patients are the best candidates for a diagnostic test. The results suggest that 5 to 10 markers will be needed to achieve optimal predictive power.

**Conclusions:**

This study shows that epigenetic field effects differ significantly between cancer patients and controls. Their detection in benign biopsy cores can form the basis of diagnostic tests to identify patients in need of repeat biopsies, reducing the cost of continued PCA screening by up to 40%. They could also be used to identify prostate cancer patients with low grade disease who are likely candidates for active surveillance or focal therapy.

**Electronic supplementary material:**

The online version of this article (doi:10.1186/s40364-014-0025-9) contains supplementary material, which is available to authorized users.

## Background

For over 2 decades, the prostate specific antigen test (PSA) has been used to screen for prostate cancer (PCA) with controversial outcomes [1-3]. Studies have shown that there is no PSA cutoff that would simultaneously optimize the sensitivity and specificity [4-8]. Lowering the PSA cutoff improves the sensitivity but drastically increases the number of unnecessary biopsies, particularly for patients with PSA levels below 10 ng/ml.

Prostate biopsies remain the gold standard for PCA diagnosis. With a sensitivity of about 70%, they miss about one third of cancers because they only sample 1% of the prostate gland [9-11]. Therefore, a negative first biopsy can’t rule out the presence of cancer. Patients at high suspicion of cancer following a first negative biopsy present a challenge to the treating physician who needs to balance the morbidity associated with repeat biopsies with the risk of missing what might be a significant cancer [12-14]. Cancers detected on first re-biopsy are not necessarily lower grade or stage which makes a repeat biopsy the safest clinical option [15,16]. Despite years of research, we still lack diagnostic methods capable of identifying patients with a negative first biopsy who are in need of a repeat biopsy with the sensitivity and specificity needed to spare non cancer patients the burden of repeat biopsies.

The detection of abnormal genetic and epigenetic fields associated with cancer in benign biopsy cores or circulating DNA could serve as a diagnostic tool to identify patients in need of repeat biopsies. Their presence has been inferred from variations in gene expression and protein levels, from somatic mutations, deletions, and DNA methylation [17-28]. The PCA3 urine test, which measures the expression levels of a prostate specific gene in cells recovered following a digital rectal exam (DRE), differentiated cancer patients from controls with 77% sensitivity and 57% specificity [29,30]. Several studies have shown that the gene expression profile of tumor-associated benign tissues differs significantly from benign tissue obtained from tumor free donors [17-19]. The analysis of negative first biopsies for the presence of a deletion in the mitochondrial genome identified patients in need of repeat biopsies with 84% sensitivity and 54% specificity [22]. None of the tests available today achieved the clinical utility needed to justify the cost of screening. Technical assessments by insurance providers also find insufficient evidence to support the use of gene-based tests for PCA diagnostics [31-33].

Epigenetic field effects in histologically normal tissues of cancerous prostates are well documented [23-28]. The MATLOC study showed that the number of unnecessary repeat biopsies can be reduced by analyzing benign cores for the methylation of 3 CpG islands commonly methylated in prostate cancer [28]. Similar to gene-based tests, studies of epigenetic biomarkers did not achieve the sensitivity and specificity needed for widespread clinical adoption. We undertook this study to identify additional epigenetic markers capable of accurately differentiating between pathologically benign and cancerous prostate tissues. We selected 19 additional markers: *ADCY4, ARHGEF10, CXCL14, CYBA, GFRA2, GPX7, GRASP, HAPLN3, HEMK1, HOXB5, HOXD9*, *KIFC2, KLK10, LOXL2, MOXD1, NEUROG3, RASSF5, SLC16A5,* and *SOCS3* (as described in Materials and Methods). We included *GSTP1, APC, PTGS2, RARB*, and *RASSF1* to facilitate comparison of our results to published reports. We analyzed the methylation of the entire panel in DNA recovered from cancer, abnormal and benign biopsy tissues obtained from patients undergoing repeat biopsies. The data shows that the methylation of benign tissues differs significantly between cancer patients and controls. Methylation is minimal in benign tissues from controls and extensive in both benign and cancer cores from cancer patients. The differential methylation of benign prostatic tissues can be exploited to improve PCA diagnostics. It can serve as the basis for a diagnostic test aimed at eliminating unnecessary biopsies which will likely require 5 to 10 markers to achieve the predictive power needed for clinical adoption. It may also be useful in the selection of patients who are likely candidates for watchful waiting or focal therapy. The number of methylated benign cores and the extent of their methylation will likely have additional diagnostic value as markers for high grade disease and poor prognosis.

## Results

### Patient characteristics

One hundred and four patients (67 controls and 37 cases) were enrolled in this retrospective study. Patient characteristics are shown in Table 1. Ninety patients underwent multiple biopsies including 66 out of 67 controls. Patients ranged in age between 49 and 86 years old. Patients with elevated PSA were considered controls if they underwent repeat biopsies and have not yet been diagnosed with prostate cancer. The follow-up time ranged from 19 to 64 months. None of the controls underwent an additional biopsy at the end of the study to rule out the presence of prostate cancer. They only received routine care.

**Table 1:** **Patient characteristics and available tissues**

**a. Prostate biopsy cores**					
**Characteristic**		**Controls (N = 67)**		**Cases (N = 37)**	
Age (yrs)		63.66 (8.1, N = 67)		70.9 (9.0, N = 31)	
Race					
Black		3 (4.5%)		1 (2.7%)	
Hispanic		0 (0%)		0 (0%)	
White		64 (95.5%)		19 (51.4%)	
Unknown		0 (0%)		17 (45.9%)	
**Number cores available**					
1		10 (14.9%)		0 (0%)	
2		56 (83.5%)		28 (75.7%)	
3		1 (1.5%)		4 (10.8%)	
4		0 (0%)		5 (13.5%)	
Total		125		88	
**Number benign cores available**					
1		25 (37.3%)		29 (78.4%)	
2		41 (61.2%)		2 (5.4%)	
3		0 (0%)		1 (2.7%)	
**Number abnormal cores available**					
1		17 (25.4%)		7 (18.9%)	
2		0 (0%)		3(8.1%)	
3		1 (1.5%)		0 (0%)	
**Number cancer cores available**					
1		0 (0%)		35 (94.6%)	
2		0 (0%)		2 (5.4%)	
**b. Patient characteristics**					
**Patient status**	**Attribute**	**Median**	**Mean**	**Std Dev**	**Range**
**Controls**					
	Age (n = 67)	63.33	63.66	8.11	49.17-86.08
	PSA (n = 66)	6.22	7.3	4.651	0.09-23.31
	Follow Up In Mos. (n = 67)	53	50.46	10.656	19-66
**Cases**					
	Age (n = 31)	72.17	70.77	9.025	52.33-84.33
	PSA (n = 29)	5.9	6.45	2.28	2.9-12
	Case Gleason Score (n = 29)	7	7.1	0.938	6-9
	Core Gleason Score (n = 38)	6	6.53	1.269	4-9
	% Core Involved (n = 38)	20	23.73	18.995	<1-80

On average, 2 biopsy cores were obtained per patient. For controls, we analyzed 2 histologically benign cores or 1 benign and 1 abnormal core (high grade prostatic intraepithelial lesions (HGPIN) or atypical small acinar proliferation (ASAP)) when available. For cancer cases, one of the 2 cores was the cancer core and the second core was either benign or abnormal. The volume of cancer was estimated by the pathologist based on 2 H&E stained sections which flanked the 5 sections used for DNA preparation. The cancer volume varied from <1% to 80% with a median of 20 and a mean of 25% (SD 18.995). The sections we received from one case were devoid of cancer but ASAP was present. For the purpose of this study, we classified the core as cancer because the sections were derived from within 10 microns of the pathologically visible cancer. This core exhibited methylation at 9 markers.

### Quantitative MS-PCR assay optimization

For this study, we selected 19 novel markers with methylation frequencies in PCA ranging between 50 and 90% and developed semi-quantitative MS-qPCR assays suitable for the analysis of formalin-fixed paraffin-embedded biopsy tissues. Marker selection and a list of CpG islands and assay conditions are described in the Additional file 1.

Genereux et al. showed that bisulfite conversion of methylated cytosines accrue on DNA templates mostly after all unmethylated cytosines have been converted to uracil [34]. To minimize failed conversions of unmethylated cytosines and reduce the false positive error rate, we introduced *in vitro* methylation at AluI (AGCT) and HaeIII (GGCC) sites on all templates prior to deamination. The *in vitro* methylation resulted in methylated cytosines at known locations in every DNA fragment which was expected to drive the preferential deamination of unmethylated cytosines.

In DNA methylation studies, the use of a reference gene to estimate the degree of deamination of a sequence of interest is commonly accepted even though there is no experimental evidence to suggest that all DNA sequences deaminate or degrade at the same rate. The use of a reference gene is valid only if it deaminated and degraded at the same rate as the sequence of interest. During assay optimization, we observed that markers deaminated and degraded at different rates that were dependent on DNA concentration. We could not use a single reference sequence to estimate the degree of deamination of all 24 markers. Instead, we determined using cancer cell line DNAs, the optimal length of bisulfite treatment for each marker and selected conditions that resulted in no detectable amplification from negative controls (lymphoblastoid DNAs and some of the cancer cell lines) and a robust amplification from positive controls (prostate cancer cell lines and fully methylated CCL119 DNA). This approach yields data that is not dependent on reference genes or clinical information for accuracy and is more suitable for the analytical validation methods required for diagnostic tests. It allows for absolute marker quantitation independent of reference genes. It also allows for the empirical determination of the range of DNA concentrations suitable for analysis under a given bisulfite conversion protocol and the limit of detection of individual marker.

For each MS-qPCR assay, we determined using control DNAs, the analytical sensitivity and specificity for increasing amounts of cancer cell line DNAs (from 0.625 ng to 20 ng) to determine the range of DNA concentrations suitable for deamination under the selected conditions. All markers were detectable from the 2.5 ng bisulfite reactions from at least 1 cancer cell line corresponding to an input of about 100 copies pre-bisulfite per multiplex PCR reaction. We used a single cutoff of 35 cycles for all MS-qPCR assays. Any sample yielding an amplification signal for any marker below 35 cycles was considered methylated at that marker. For markers *RASSF5, MOXD1, KIFC2, NEUROG3*, and *HEMK1*, two assays from different parts of the CpG islands or from opposing strands were included and the data was pooled for statistical analysis.

We collected 5076 data points for 24 markers from 213 cores, 2991 data points from 125 control cores and 2085 from 88 case cores. For the control cores, 2803 out of 2991 (93.7%) yielded no detectable amplification (no rise in fluorescence level above background). For the cancer cases, 1263 reactions out of 2085 yielded no amplification, 866 from 49 benign/abnormal cores (73.6%) and 397 from 39 cancer cores (42.4%).

### Marker characteristics in patients with elevated PSA

The estimated clinical sensitivity and specificity for the presence (>0 methylation level) of each marker were computed using cancerous cores from cases and the core with the highest number of methylated markers for controls (Table 2). We discarded the data for the remaining control cores to avoid biasing the specificity estimates by representing each control twice. We chose the most methylated core because it represents the highest level of methylation available for the individual. The observed sensitivity for the 24 markers ranged between 11 and 97%, while the specificity ranged between 66 and 100% with the majority of markers exceeding 90%. Overall, we observed low levels of methylation in cores derived from non-cancerous prostates. The sensitivities and specificities reported here were for the assays that we chose. For some markers like *GSTP1* and *APC*, we tested multiple primer-probe combinations before selecting the best performing assay for FFPE biopsy DNA. For other markers like *ARHGEF10* and *KLK10*, we only tested a single probe. The sensitivities and specificities of some markers could be further improved by testing different primers/probe combinations or further optimizing the bisulfite conditions.

**Table 2:** **Sensitivities and specificities for individual markers and combination of markers**

	***Cancer cores***			***Control cores***			***Non cancer cores from cases***		
***Marker***	***No. Pos/No. cases***	***Sensitivity***	***95% CI***	***No. Neg/No. controls***	***Specificity***	***95% CI***	***No. Pos/No. cases***	***Sensitivity***	***95% CI***
CYBA	22/37	0.59	(0.44, 0.75)	64/67	0.96	(0.91, 1.00)	5/37	0.14	(0.02, 0.25)
HOXB5	31/37	0.84	(0.72, 0.96)	56/67	0.84	(0.75, 0.92)	24/36	0.67	(0.51, 0.82)
RASSF1	33/37	0.89	(0.79, 0.99)	55/67	0.82	(0.73, 0.91)	16/37	0.43	(0.27, 0.59)
SOCS3	27/37	0.73	(0.59, 0.87)	61/67	0.91	(0.84, 0.98)	17/36	0.47	(0.31, 0.64)
GRASP	22/37	0.59	(0.44, 0.75)	64/67	0.96	(0.91, 1.00)	1/37	0.03	(0.00, 0.08)
HAPLN3	27/37	0.73	(0.59, 0.87)	63/67	0.94	(0.88, 1.00)	11/37	0.30	(0.15, 0.44)
SLC16A5	11/37	0.30	(0.15, 0.44)	64/67	0.96	(0.91, 1.00)	2/37	0.05	(0.00, 0.13)
HOXD9	34/37	0.92	(0.83, 1.01)	43/65	0.66	(0.55, 0.78)	29/36	0.81	(0.68, 0.93)
ARHGEF10	8/37	0.22	(0.08, 0.35)	62/67	0.93	(0.86, 0.99)	7/37	0.19	(0.06, 0.32)
KLK10	14/37	0.38	(0.22, 0.53)	67/67	1.00	--	1/37	0.03	(0.00, 0.08)
GSTP1	25/36	0.69	(0.54, 0.84)	64/67	0.96	(0.91, 1.00)	10/37	0.27	(0.13, 0.41)
RASSF5	28/37	0.76	(0.62, 0.90)	61/67	0.91	(0.84, 0.98)	21/37	0.57	(0.41, 0.73)
MOXD1	12/37	0.32	(0.17, 0.48)	61/67	0.91	(0.84, 0.98)	6/37	0.16	(0.04, 0.28)
RARB	25/37	0.68	(0.52, 0.83)	64/67	0.96	(0.91, 1.00)	4/37	0.11	(0.01, 0.21)
GPX7b	16/37	0.43	(0.27, 0.59)	64/67	0.96	(0.91, 1.00)	5/37	0.14	(0.02, 0.25)
APC	27/36	0.75	(0.61, 0.89)	61/67	0.91	(0.84, 0.98)	12/35	0.34	(0.19, 0.50)
GFRA2	13/36	0.36	(0.20, 0.52)	64/67	0.96	(0.91, 1.00)	5/35	0.14	(0.03, 0.26)
LOXL2	11/36	0.31	(0.16, 0.46)	65/67	0.97	(0.93, 1.00)	5/35	0.14	(0.03, 0.26)
NEUROG3	18/36	0.50	(0.34, 0.66)	64/67	0.96	(0.91, 1.00)	6/35	0.17	(0.05, 0.30)
PTGS2	4/36	0.11	(0.01, 0.21)	65/67	0.97	(0.93, 1.00)	10/35	0.29	(0.14, 0.44)
ADCY4	36/37	0.97	(0.92, 1.03)	56/67	0.84	(0.75, 0.92)	20/37	0.54	(0.38, 0.70)
CXCL14	27/37	0.73	(0.59, 0.87)	63/67	0.94	(0.88, 1.00)	10/37	0.27	(0.13, 0.41)
HEMK1	15/37	0.41	(0.25, 0.56)	60/67	0.90	(0.82, 0.97)	2/37	0.05	(0.00, 0.13)
KIFC2	22/37	0.59	(0.44, 0.75)	67/67	1.00	--	8/37	0.22	(0.08, 0.35)
3 of 24	37/37	1.00	--	46/67	0.69	(0.58, 0.80)	31/37	0.84	(0.72, 0.96)
4 of 24	37/37	1.00	--	56/67	0.84	(0.75, 0.92)	23/37	0.62	(0.47, 0.78)
5 of 24	37/37	1.00	--	65/67	0.97	(0.93, 1.01)	23/37	0.62	(0.47, 0.78)
6 of 24	36/37	0.97	(0.92, 1.03)	66/67	0.99	(0.96, 1.01)	19/37	0.51	(0.35, 0.67)
7 of 24	36/37	0.97	(0.92, 1.03)	66/67	0.99	(0.96, 1.01)	18/37	0.49	(0.33, 0.65)
8 of 24	36/37	0.97	(0.92, 1.03)	67/67	1.00	--	15/37	0.41	(0.25, 0.56)
9 of 24	35/37	0.95	(0.87, 1.02)	67/67	1.00	--	11/37	0.30	(0.15, 0.44)
10 of 24	30/37	0.81	(0.68, 0.94)	67/67	1.00	--	11/37	0.30	(0.15, 0.44)
11 of 24	27/37	0.73	(0.59, 0.87)	67/67	1.00	--	5/37	0.14	(0.02, 0.25)
12 of 24	25/37	0.68	(0.52, 0.83)	67/67	1.00	--	5/37	0.14	(0.02, 0.25)
13 of 24	20/37	0.54	(0.38, 0.70)	67/67	1.00	--	3/37	0.08	(0.00, 0.17)
14 of 24	17/37	0.46	(0.30, 0.62)	67/67	1.00	--	2/37	0.05	(0.00, 0.13)
15 of 24	15/37	0.41	(0.25, 0.56)	67/67	1.00	--	1/37	0.03	(0.00, 0.08)
16 of 24	14/37	0.38	(0.22, 0.53)	67/67	1.00	--	0/37	0.00	--
17 of 24	11/37	0.30	(0.15, 0.44)	67/67	1.00	--	0/37	0.00	--
18 of 24	7/37	0.19	(0.06, 0.32)	67/67	1.00	--	0/37	0.00	--
19 of 24	7/37	0.19	(0.06, 0.32)	67/67	1.00	--	0/37	0.00	--
20 of 24	3/37	0.08	(0.00, 0.17)	67/67	1.00	--	0/37	0.00	--

Shows the estimated sensitivity and specificity associated with each marker (where a marker test is defined as the presence (>0 concentration) or absence (0 concentration) of the particular marker). The column preceding sensitivity yields the number of “positive tests” and the number of “true cases”. Similarly, the column preceding specificity yields the number of “negative tests” and the number of “true controls”. Similarly for the non-cancer cores from cases, the estimated sensitivity associated with each marker and the sensitivity associated with the total number of markers were calculated using the non-CA core with the highest number of positive markers from each case.

### Methylation is cumulative in cancer cores

Giving equal weight to all markers, we estimated the sensitivity and specificity associated with the total number of positive markers (Table 2) again using the cancer cores from cancer cases and only the most methylated core from controls. The majority of cancer cores were methylated at 9 or more markers out of the 24 tested, a level of methylation that was not observed in controls.

Table 3 presents the best 1, 2, and 3 marker predictive models observed in the sample as estimated using logistic regression with an indicator of methylation (>0) or a level of methylation for each marker included as covariates. We split the data into 2 sets, a training set and a test set. Results depict the cross-validated area under the ROC curve (AUC) on the training data and the observed AUC on test data not used in model building. Models involving *KIFC2* and *ADCY4* tended to have high out of sample diagnostic capability, with the AUC in the test data ranging from 0.91 including only *ADCY4* to 0.98 when also including *KIFC2*. Models which included the actual level of methylation were also considered and demonstrated similar results.

**Table 3:** **AUCs for training and test data**

***Without methylation level as a covariate***			***With methylation level as a covariate***		
***Model***	***Area under ROC using training data (44 controls; 24 cases)***	***Area under ROC using test Data (23 controls; 13 cases)***	***Model***	***Area under ROC using training data (44 controls; 24 cases)***	***Area under ROC using test data (23 controls; 13 cases)***
**1 Covariate models**			**1 Covariate models**		
**HOXD9**	0.82	0.74	** RASSF5**	0.84	0.88
**CXCL14**	0.82	0.86	** HOXD9**	0.88	0.92
**APC**	0.87	0.76	** APC**	0.88	0.77
**RASSF1**	0.87	0.83	** RASSF1**	0.91	0.91
**ADCY4**	0.9	0.91	** ADCY4**	0.96	0.98
**2 Covariate models**			**2 Covariate models**		
**ADCY4 + KIFC2**	0.97	0.97	** ADCY4 + LOXL2**	0.98	0.98
**RASSF1 + ADCY4**	0.96	0.95	** ADCY4 + age**	0.98	0.98
**APC + ADCY4**	0.96	0.97	** ADCY4 + RASSF1**	0.99	0.97
**RASSF1 + APC**	0.97	0.89	** ADCY4 + SOCS3**	0.98	0.99
**RASSF5 + ADCY4**	0.96	0.96	** ADCY4 + KIFC2**	0.99	0.99
**3 Covariate models**			**3 Covariate models**		
**KIFC2 + RASSF5 + HAPLN3**	0.99	0.97	** KIFC2 + CXCL14 + GSTP1**	0.99	0.94
**HOXB5 + APC + RASSF1**	0.99	0.98	** KIFC2 + ADCY4 + PTGS2**	0.99	0.99
**ADCY4 + KIFC2 + HAPLN3**	0.99	0.98	** CYBA + ADCY4 + KIFC2**	0.99	0.99
**APC + RASSF5 + CXCL14**	0.99	0.98	** KIFC2 + ADCY4 + CXCL14**	1.00	0.99
**KIFC2 + RASSF5 + CXCL14**	0.99	0.98	** SOCS3 + APC + RARB**	1.00	0.94

Shows the area under the ROC based upon training data and test data using varying degrees of model complexity. Models consider a yes/no indicator for methylation (>0) of each individual marker or use the level of methylation as a covariate.

**Figure 1:**
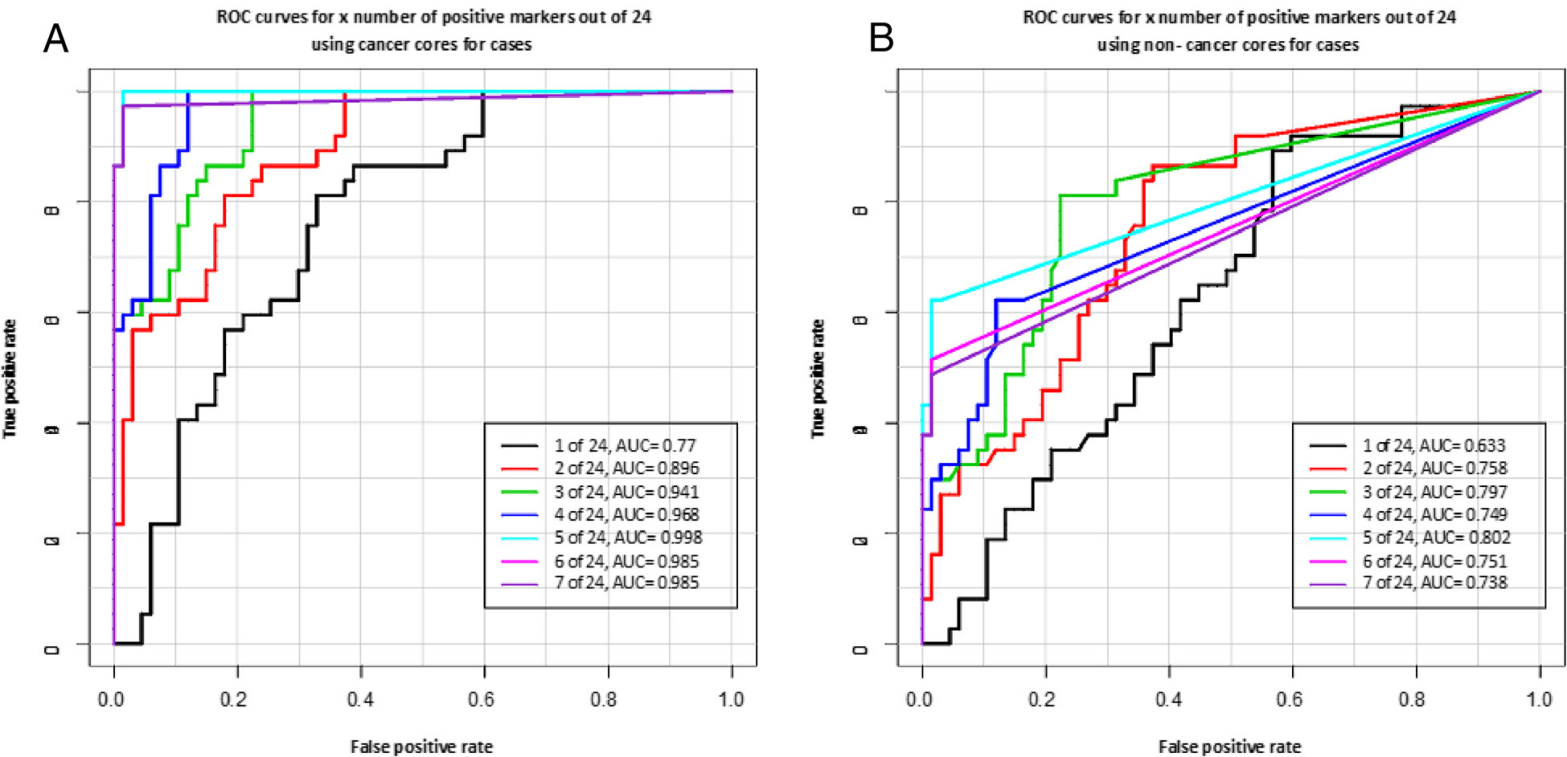
**Receiver operating characteristics (ROC) curves based on the number of methylated markers and their methylation levels generated with the data used to estimate the sensitivity and specificity in Table** **2** **. (A)** shows the ROC curves obtained when the methylation data for the cancer cores and the methylation data of the most methylated core from controls were used. **(B)** shows the ROC curves when the data for the cancer cores is replaced with the methylation of the CCNC core for each cancer patient. In cases where more than one CCNC core was available, the most methylated core was selected for analysis. Using 5 positive markers out of 24 to identify a cancer case yielded AUCs of 0.998 and 0.802 for cancer and CCNC cores, respectively.

**Figure 2:**
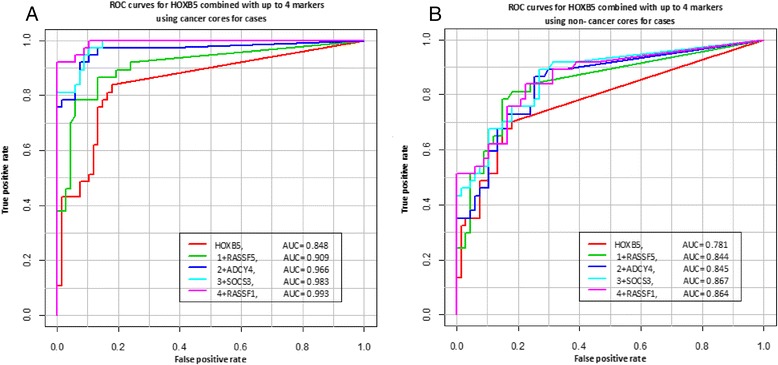
**Examples of receiver operating characteristics (ROC) curves obtained with markers that were commonly methylated in CCNC cores.** The data is shown for HOXB5, and then HOXB5 combined sequentially with up to 4 markers as shown in the list above. **(A)** shows the ROC curve obtained when the methylation data for the cancer cores and the methylation data of the most methylated core from controls were used. **(B)** shows the ROC curve when the data for the cancer cores is replaced with the methylation of the CCNC core for each cancer patient. In cases where more than one CCNC core was available, the most methylated core was selected for analysis.

Figure 1A shows the receiver operating characteristics (ROC) curves based on the total number of methylated markers and their average level of methylation for 37 cancer cores and 67 controls. As in Table 2, we used the most methylated core from controls. Using 5 positive markers out of 24 to define a cancer case yields an AUC = 0.998. Figure 2A shows the ROC curves using 5 markers commonly methylated in benign cores from cases that could potentially be used for a diagnostic test on negative first biopsies (*HOXB5, HOXB5* plus *RASSF5, HOXB5* plus *RASSF5* and *ADCY4, HOXB5* plus *RASSF5, ADCY4* and *SOCS3, HOXB5* plus *RASSF5, ADCY4, SOCS3* and *RASSF1*). The AUCs ranged from 0.848 for *HOXB5* to 0.993 when all 5 markers are combined.

### Methylation of abnormal cores

Forty seven abnormal cores harboring HGPIN or ASAP were available for this study, 20 from controls and 27 from cases, including 14 cores from additional cancer cases which were not included in the primary statistical analysis because we were unable to obtain tissues from the corresponding cancer cores. As possible precursors to cancer [35-38], we expected the abnormal cores to exhibit a methylation profile that falls between benign and cancer cores. To determine if methylation is elevated in abnormal cores, we first compared, using the two-sample t-test, the number of methylated markers in abnormal and benign cores from controls and found no significant difference between the two means (data not shown). Repeating the analysis using abnormal cores from cases and controls we found a mean difference of 2.69 (95% CI: 1.63, 3.74; p < 0.001) with abnormal cores from cases averaging 4.36 methylated markers out of 24. We then compared the methylation of abnormal and benign cores from cancer cases and found higher levels of methylation in the benign cores (mean in benign cores: 6.25, n = 36; mean in abnormal cores: 4.36 n = 27; difference in means =1.89, 95% CI: 0.24-3.54, p = 0.025). For the cores we analyzed, the results show that the methylation of abnormal cores from cases was not higher than that of the corresponding benign cores.

### Elevated methylation of normal epithelium in cancerous prostate

The non-cancer cores from cases (CCNC) were randomly chosen by the pathologist as histologically benign or abnormal. Since we were only analyzing one CCNC core from most cancer cases, we did not expect a significant difference in the methylation of benign cores between cases and controls. However, we found elevated methylation levels in over half of the 49 CCNC cores analyzed. The mean number of methylated markers in control cores was 1.5 (SD: 1.44, range: 0 to 7, n = 125 cores), 5.9 for CCNC cores (SD: 3.7, range: 0 to 15, n = 49 cores), and 13.66 for cancer cores (SD: 4.19, range: 5 to 22, n = 39 cores). Over 60% of the CCNC cores were methylated at 5 or more markers while only 3% of the control cores exhibited the same level of methylation. CCNC cores had on average 4.39 more methylated markers than control cores (95% CI: 3.06, 5.83; p < 0.001) despite being histologically equivalent.

**Figure 3:**
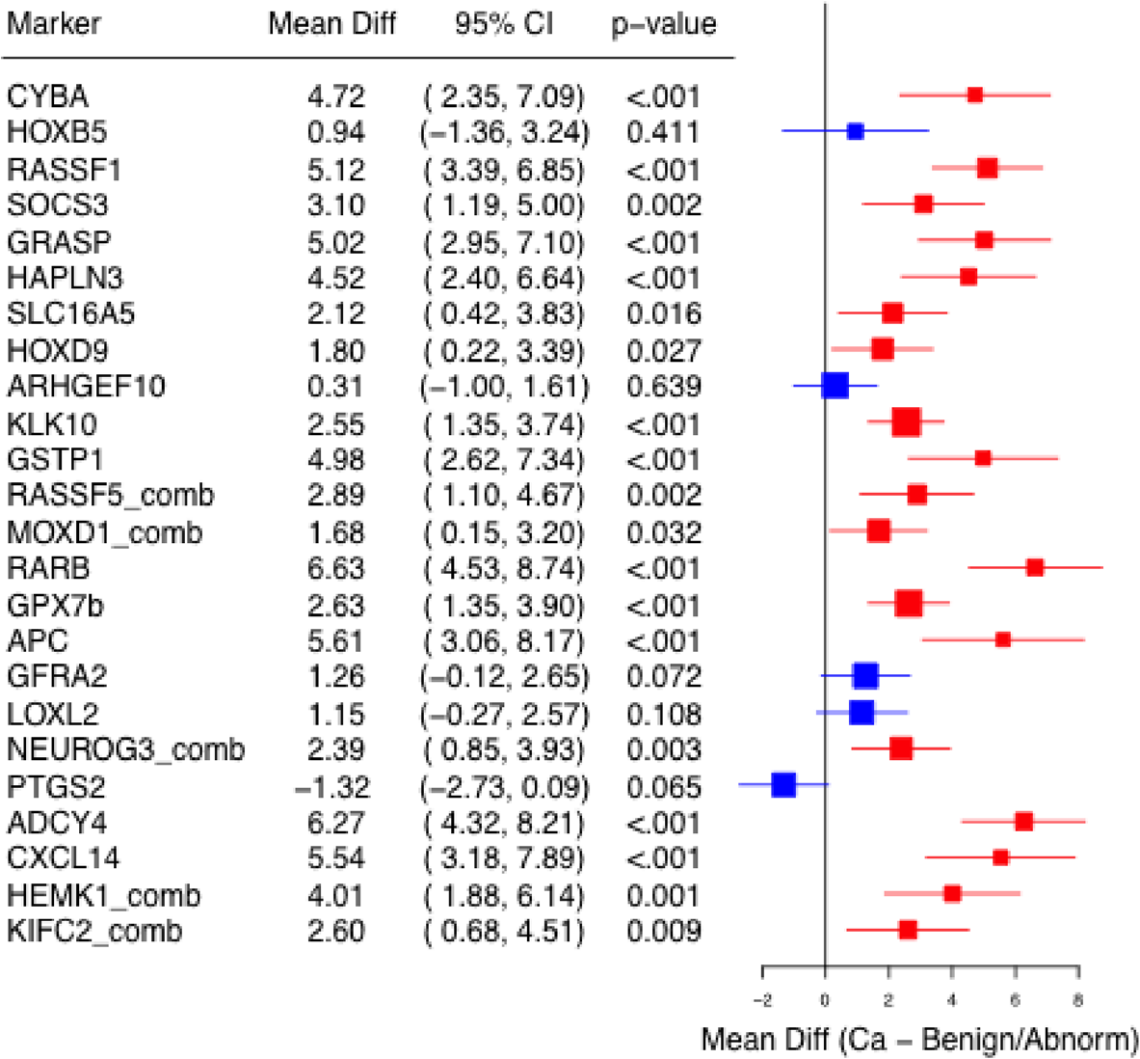
**Paired tests of mean methylation levels between cancerous and non-cancerous cores within the same case (N = 37).**

CCNC cores were also less methylated than the cancer cores even though the number of methylated markers overlapped considerably between the two. Cancer cores had on average, 7.76 more methylated markers than CCNC cores (95% CI: 5.43, 9.22; p < 0.001). Figure 3 shows the within subject mean difference in methylation levels for individual markers observed in cancerous cores when compared to benign cores among cases (N = 37). As can be seen, the observed methylation levels were consistently higher among cancerous cores when compared to CCNC cores for the majority of markers. Only *PTGS2* which codes for the cyclooxygenase 2 (*COX2*) enzyme showed higher methylation levels in CCNC cores (p < 0.065).

We repeated the statistical analysis to estimate the clinical sensitivity and specificity for individual markers using only the CCNC cores from cases (excluding all cancer cores). The results are shown in Table 2. Using the methylation of any 5 out of 24 markers in CCNC cores to define a cancer case identified 62% of cases (Table 2). The receiver operating characteristic (ROC) curve shows AUCs up to 0.802 for CCNC cores when the total number of methylated markers and their average level of methylation were considered (Figure 1B). The positive predictive value (PPV) for the CCNC cores was 89.9% (95% CI: 68.9-98.4) and the negative predictive value (NPV) was 85.7% (95% CI: 76.2-92.4) with a p < 0.0001 when a cancer case is defined by the methylation at 5 out of 24 markers. Individual markers like *RASSF5* and *ADCY4* also performed well in this test but not as well as combining the methylation content of multiple markers. Repeating the ROC curve analysis with 5 markers commonly methylated in benign cores yields an AUC of 0.781 for *HOXB5* to an AUC of 0.864 when all 5 markers (*HOXB5, RASSF5, ADCY4, SOCS3,* and *RASSF1*) are combined (Figure 2B).

### Effect of patient and core age on level of methylation

We were not able to obtain sufficient cancer cores from older cases and had to include cores that were processed more recently to obtain the 37 cases. While we expected decreased detection of methylation in older cores, this was not observed among either cases or controls. Among cases, it was estimated that a one year increase in the age of the core is associated with an average decrease of 0.64 in the number of methylated markers (95% CI: −1.47, 0.19; p = 0.127) which is not statistically significant.

We also expected an increase in methylation levels in cores obtained from older patients. However, no significant association between patient age and the total number of methylated markers was observed in either cases or controls. Among cases, it was estimated that a one year increase in age is associated with an average increase of 0.06 in the number of methylated markers (95% CI: −0.13, 0.24; p = 0.537). Similarly among controls, it was estimated that a one year increase in age is associated with an average increase of 0.03 in the number of methylated markers (95% CI: −0.01, 0.07; p = 0.537).

### Effects of tumor volume and Gleason score on density of methylation

The volume of cancer was less than 20% in 24 cores and greater than 30% in 15 cores. Twenty one cancer cores had a Gleason score of 6 or lower and 17 had a Gleason score of 7 or higher. For most cores, the cancer cells did not contribute enough DNA to be solely responsible for the methylation detected with these markers. We expect that the epigenetic field which extends beyond pathologically defined cancer contributed significantly to the methylation pattern. We also did not see any correlation between the methylation density and the Gleason score of the core analyzed (data not shown). However, we analyzed a relatively small number of cores with a wide range of tumor volumes using only 24 markers. Correlations between methylation density, tumor grade and volume are best done with properly matched cores to avoid errors introduced by the variable number of cancer cells. If the trend we observed continues in larger studies and with additional markers, methylation may become an additional variable independent of pathology that can be used to stratify patients.

## Discussion

Epigenetic field effects have previously been reported in prostate cancer. Truong et al. [24] detected DNA methylation in benign tissues distant from tumors while Stewart et al. [28] showed that the methylation can be used to reduce the number of unnecessary repeat biopsies. Our study also supports the presence of an epigenetic field effect that is not in direct proximity to cancer foci. The non-cancer cores we analyzed were randomly chosen by the pathologist and were not selected from close proximity to known cancer cores. While we cannot rule out that all methylation positive CCNC cores were derived from near cancer lesions, this possibility is highly unlikely since the majority of cores were from repeat biopsies. The likelihood that the benign cores harbored small cancer foci that were not detected during three pathological reviews is also unlikely because 10 to 25% of the cells have to be methylated in order for most markers to be detected using our assays. It is more likely that the markers are identifying precancerous or cancerous lesions that aren’t yet pathologically identifiable. Our data supports the presence of multiple molecularly abnormal but histologically normal lesions in cancerous prostates which are not present or present to a significantly lesser extent in non-cancerous prostates. They may contribute to the multifocal nature of prostate cancer.

Abnormal cores from cancer cases did not exhibit higher methylation levels than the benign cores for the 24 markers analyzed. Overall, the observed methylation does not support a role for abnormal cores as precursors to cancer even though we cannot rule out that the amount of DNA contributed by the abnormal cells was below the limit of detection. It is highly possible that the methylation of the abnormal cores was similar to the methylation of benign cores because the benign cells present in abnormal cores contributed the methylation pattern and the presence of histological aberrations was a coincidental occurrence. It is also possible that the epigenetic signature of abnormal cores differs significantly from cancer cores and could not be detected using cancer specific markers.

We selected a 24 marker panel for this study because we were expecting significant overlap in methylation levels between cancer cases and controls as previously reported [24-28]. We intended to identify the best performing markers for further development. However, this study showed that there is minimal overall methylation in controls. Methylation accumulated only in cancerous prostates which supports the involvement of all selected markers in the transformation process. ROC curve analysis (Figures 1b and 2b) supports the selection of a limited number of markers commonly methylated in CCNC cores for the development of an epigenetic test aimed at eliminating unnecessary repeat biopsies. *HOXB5, RASSF5, ADCY4, SOCS3, and RASSF1* (Figure 2b) yielded better AUCs than all 24 markers combined (Figure 1b).

We were able to identify 62% of cancer cases based on the methylation pattern of a single randomly chosen CCNC core which raises the question of how widespread the epigenetic field effect is in prostate cancer. It is difficult to estimate the number of benign cores that are methylated in cancerous prostates from our study because we analyzed an average of 1.3 benign cores per case from patients undergoing repeat biopsies. However, we anticipate that the number of positive cores will follow a normal distribution heavily skewed towards zero in non-cancerous prostates and centering around 5 or 6 cores in cancerous prostates. We also anticipate that the number of methylated markers per core will follow a normal distribution. The combination of the number of methylated markers and cores can be used to map all abnormal areas over the entire gland and convert the methylation map into risk scores to classify men based on the likelihood of a positive diagnosis.

The number of methylated markers in CCNC cores ranged between 0 and 15 and overlapped considerably with cancer cores. In some cases, the CCNC core was more methylated than the cancer core. Some markers like *HOXB5, RASSF5, ADCY4, SOCS3*, and *RASSF1* were frequently methylated in CCNC cores and may represent early epigenetic events in tumorigenesis. Others like *GRASP, HEMK1, RARB* and *SLC16A5* were methylated mostly in histologically detectable cancer and may represent later events. The differential methylation of CCNC and cancer cores may identify critical genes and pathways that are required for the development of histologically visible cancer. It may also reflect the order of acquisition of methylation events and help define a molecular clock for PCA.

Epigenetic mapping of the prostate gland may also be useful in patients with low risk disease who choose a more conservative treatment approach. Over one third of men under active surveillance for low grade prostate cancer are upgraded within 5 years because of disease progression [39,40]. Mapping of DNA methylation in benign epithelium may help identify men who are at higher risk for disease progression at the time of first biopsy. For this purpose, a larger panel of markers may be needed that includes markers preferentially methylated in cancer cores in addition to markers commonly methylated in CCNC cores. We were able to detect methylation in several patients with negative biopsies up to 4 years prior to a cancer diagnosis (data not shown) which suggests that the epigenetic aberrations are present at least 4 years prior to a positive biopsy. The ability to accurately map in biopsy cores the number of abnormal fields and the extent of their methylation may help identify patients with low grade disease who are not good candidates for active surveillance.

To date, PCA screening and early clinical intervention while prostate cancer is organ confined haven’t led to a significant reduction in mortality [41,42]. Epigenetic aberrations can be detected prior to the development of histologically visible cancer. This may allow for earlier clinical intervention in men at high risk for aggressive disease. It may also improve the timing and selection of treatment for men with slower growing cancers reducing overtreatment of cancers that are clinically insignificant.

*PTGS2* was the only marker out of the 24 analyzed that was more frequently methylated in CCNC than in cancer cores. The *PTGS*2 gene codes for the COX2 enzyme which is the target of inhibition by aspirin. Epidemiological studies have shown that long term aspirin use is potentially associated with a modest reduction in prostate cancer risk [43,44]. The inactivation of the *PTGS2* promoter may slow the growth of precancerous lesions and reduce the likelihood of progression to pathologically detectable cancer. CCNC cores may harbor additional methylation that reduces the growth potential of abnormal cells. There may be value in mapping epigenetic events that accumulate preferentially in CCNC cores to identify pathways that can be targeted for prostate cancer prevention or treatment.

The MATLOC study analyzed the CpG islands associated with *GSTP1*, *APC*, and *RASSF1* in benign biopsy cores from 498 patients undergoing repeat biopsies [28]. The 3 marker combination yielded 68% sensitivity and 64% specificity based on the methylation of 10 or more biopsy cores. In our study, these 3 markers yielded 48% sensitivity, 89% specificity and an AUC of 0.731 based on the analysis of a single CCNC core. Direct comparison between the 2 studies is not straightforward because of the difference in assay methodology, patient population, and number of cores analyzed. However, based on the data collected from all 125 benign cores from controls, we expect the specificity of these 3 markers to be significantly higher than the 64% reported by MATLOC. The higher specificity observed in our study is likely due to the bisulfite conversion conditions which were tailored for each marker, and were different for *RASSF1* than for *APC* and *GSTP1*. The majority of cases in the MATLOC study showed methylation at 2 cores or less. Given that we identified 62% of cases from the methylation of a single core, we expect the average number of positive cores per case to be significantly higher. Additional studies on larger cohorts are needed to better determine the performance of these 3 markers and the rest of the markers presented here.

Several studies have associated field effects in prostate with the likelihood of higher grade disease. Makarov et al. [19] showed that the levels of proPSA in tumor adjacent tissues at the time of biopsy are associated with the need for further treatment in patients enrolled in expectant management for PCA. Veltri et al. [45] showed that the nuclear structure information of benign tumor-associated tissues can help predict the likelihood of metastatic progression. Could the epigenetic aberrations in a cancerous prostate also help determine the likelihood of clinically significant disease at the time of diagnosis? Furthermore, do aberrant epigenetic fields correlate or even contribute to cancer metastasis? The first question can easily be answered by correlating the methylation of prostatectomy specimens with clinical outcomes. The second is more difficult because metastatic tissue samples are difficult to obtain. However, circulating metastatic cells and cell free DNA may be an acceptable substitute particularly as improved molecular methods require significantly smaller amounts of starting material.

The extensive methylation in CCNC cores raises many important questions that could help elucidate the etiology of prostate cancer. If the differential methylation between controls and cases is validated in larger studies, it may point to an infectious agent underlying the initiation of prostatic carcinogenesis. Several studies have linked subtypes of *Propionibacterium acnes* to prostate inflammation and suggested a potential role for *P. acnes* as a carcinogenic infectious agent [46-48]. Future prospective studies evaluating DNA methylation could also investigate if *P. acnes* co-localizes with DNA methylation in cancerous prostates.

Approximately 50% of men with a first negative biopsy will continue to have elevated PSA on subsequent screening [49]. If half elect to undergo additional biopsies at an average cost of $3,172 [50], the repeat biopsy cost will exceed $1 billion per year. To be widely adopted, a diagnostic test to eliminate unnecessary biopsies needs to balance the clinical utility with the cost of screening. A diagnostic test that costs as much as a prostate biopsy will not be readily adopted because it increases diagnostic costs as positive molecular findings will trigger a repeat biopsy for 30% of patients or more.

We anticipate that a diagnostic test for patients in need of a repeat biopsy will require the analysis of 5 to 10 markers on all available cores for optimal predictive power. For each biopsy, twelve or more cores will be analyzed. Each additional marker will significantly impact the cost and the complexity of the detection assays. It would be cost effective to minimize the number of assays by selecting the most informative and the most analytically robust markers that can be amplified in a single multiplex. However, this study clearly demonstrates the impact of combining the information from multiple markers. There is significant predictive power in the number of methylated cores and the number of methylated markers per core that can be translated into clinical utility. Further studies of these and additional markers are needed to identify the optimal marker panel that would maximize clinical value while minimizing screening costs. If the methylation of first negative biopsies can be analyzed for about $1,000, an epigenetic test could reduce the cost of continued PCA screening by over 40% eliminating up to 70% of repeat biopsies.

Epigenetic field effects have been documented in many cancers [51]. Their role in tumorigenesis is not yet well understood. The results of this study suggest that more than 24 markers will be needed to understand the contribution of aberrant fields to the initiation and progression of cancer. Our ability to detect them in pathologically benign epithelium with improved analytical accuracy could have a profound impact on cancer diagnostics, treatment selection, and patient management.

## Conclusion

In this study, we presented a panel of 24 markers capable of differentiating between prostate cancer and benign tissues with greater than 97% sensitivity and specificity. To the best of our knowledge, this is the first study to integrate the methylation information from 24 markers in prostate biopsy tissues and show significant differences in the methylation of benign cores from PCA patients and controls. This study supports the development of a biopsy-based epigenetic test to reduce the cost of continued screening for PCA. Our data suggests that 5 to 10 markers will be required to achieve optimal predictive power. The detection and quantitation of epigenetic fields may have additional applications in identifying prostate cancer patients who are likely candidates for active surveillance or patients who are at higher risk for aggressive cancer and progression.

## Materials and methods

Please see the Additional file 1 for details on marker selection and assay optimization.

### Biopsy tissues

Formalin-fixed paraffin-embedded biopsy tissues (FFPE) from 100 patients were provided by Dr. Daniel Murtagh (Promedica Genitourinary Surgeons, Toledo OH) under an IRB protocol approved by Western Institutional Review Board (WIRB, study #1123188, Puyallup, WA). The tissue samples were previously processed as part of routine care in a single clinic in Ohio. An additional 4 cancer cases were provided by StrataDX (Lexington, MA). All patients were deidentified so only the referring physician and pathologist knew their identity. Biopsy cores were obtained from 37 cancers and 67 controls. For each case, we obtained on average 2 cores. For the control cases, either both cores were benign or one of the cores harbored abnormalities such as high grade intraepithelial neoplasia (HGPIN) or atypical small acinar proliferation (ASAP). For the cancer cases, one of the cores was cancer or immediately adjacent to cancer (for 1 case). For 2 cases, the cancer core contained less than 1% cancer by volume. Additional cores from cancer cases were benign or abnormal. We analyzed 125 cores from control cases and 88 cores from cancer cases. For each core, we obtained 5 unstained 8 micron sections for DNA extraction. The laboratory personnel were blinded to the pathological diagnosis until after the marker data was collected.

Twenty abnormal cores from controls and 13 from cases harbored pathological abnormalities such as HGPIN or ASAP. An additional 14 abnormal cores were available from first negative biopsies from cancer patients with follow-up time to a positive diagnosis ranging between 2 and 46 months. We were unable to obtain the positive biopsy for these 14 cases. Their data was included only in the analysis of methylation of abnormal cores.

### Statistical methods

Patient characteristics were summarized via the arithmetic mean and standard deviation for continuous characteristics, and frequency and percentage for categorical characteristics. Within-subject differences in mean methylation levels between cancerous and non-cancerous core samples were tested with the paired t test, while mean differences in methylation levels of non-cancerous cores among cases were compared to controls using the two-sample t-test with unequal variances. The associations between patient and core sample age with methylation level were quantified using linear regression stratified by patient disease status. Sensitivity and specificity associated with the presence of individual markers, and the total sum of positive markers, were computed using the observed proportion of individuals with positive markers conditional upon true disease status. Corresponding 95% confidence intervals for sensitivity and specificity were computed using the normal approximation to the binomial distribution with continuity correction. Multiple logistic regressions were used to estimate diagnostic risk scores based upon multiple markers. To develop multiple marker risk scores, the data were randomly divided into two parts. The first part was used for model training and assessment (two thirds of the available data; 44 controls and 24 cases), and the second was used for model testing (one third of available data; 23 controls and 13 cases). The test data set was not used in model fitting and selection. A best subsets procedure was used to identify the top diagnostic models using data from multiple markers. Specifically, we considered all possible one, two, and three marker combinations. Top performing models were selected based upon the area under the receiver operating characteristic curve (AUC) that was observed using the risk score defined by the linear predictor from the logistic regression model for the considered covariates. Four-fold cross validation estimates of AUC were used to rank models for their predictive ability while avoiding over-fitting at the training stage. The top performing models (those attaining the highest AUC in the training data) where then applied to the test data. Parameter estimates were not re-estimated using the test data, but were maintained from the training data in order to provide an honest assessment of out-of-sample diagnostic performance. After applying each model to the test data, risk scores were generated and the AUC for the test sample was computed. AUC was computed using the ROCR package (http://cran.r-project.org/web/packages/ROCR) for the R statistical programming language (Ver 3.0). Models including only a binary indicator for methylation level (>0 vs 0) as well as the methylation level were considered. To calculate the mean difference in the number of methylated markers between 2 groups of biopsy cores, we used the two-sample t-test function.

## Additional file

Additional file 1:
**Contains the methods used to generate the marker data.** It shows the marker selection, the chromosomal location of CpG islands, and a list of the primers and conditions used for MS-qPCR assays. It also describes the optimization protocol used for the bisulfite conversion [52-57].

## Abbreviations

PCA: Prostate cancer
PSA: Prostate specific antigen
BPH: Benign prostatic hyperplasia
FFPE: Formalin-fixed paraffin embedded
HGPIN: High grade prostatic intraepithelial lesions
ASAP: Atypical small acinar proliferation
MS-qPCR: Methylation-specific quantitative polymerase chain reaction
ROC: Receiver operating characteristic
AUC: Area under curve
UTR: Untranslated region
CCNC: Non-cancer cores from cancer cases
DRE: Digital rectal exam
