# Supplementary material for: A panel of DNA methylation markers reveals extensive methylation in histologically benign prostate biopsy cores from cancer patients
Source: Biomark Res. 2014 Dec 12;2:25. doi: 10.1186/s40364-014-0025-9 (PMC4278343; doi:10.1186/s40364-014-0025-9)
Supplement: Additional file 1: — Contains the methods used to generate the marker data. It shows the marker selection, the chromosomal location of CpG islands, and a list of the primers and conditions used for MS-qPCR assays. It also describes the optimization protocol used for the bisulfite conversion [52-57]. [file 40364_2014_25_MOESM1_ESM.pdf]

Materials and Methods

DNA preparation: DNA was extracted from paraffin-embedded tissues using an extended proteinase K (Affymetrix) extraction and recovered in TE8 following ethanol precipitation. DNA was quantitated using the Qubit fluorometer (Life Technologies, Grand Island, NY) using a DNA quantitation kit (Life Technologies, Kit # Q32854). Samples with less than 20 ng of DNA were excluded from analysis. The cancer cell lines DU145 (ATCC HTB-81D, prostate cancer), PC3 (ATCC CRL-1435D, prostate cancer), CCRF-CEM (ATCC CCL-119, leukemia), and COLO-205 (ATCC CCL-222, colon cancer) were used as controls. DNAs and cell lines were obtained from ATCC.

Determination of optimal bisulfite conversion conditions:

To determine the optimal bisulfite deamination conditions for each marker, we initially performed a time course using sodium bisulfite conversion which showed that markers deaminate and degrade at different rates influenced by DNA concentration and methylation status. The markers we selected would have required sodium bisulfite treatments ranging between 6 and 18 hours. A single deamination condition wouldn't have yielded the correct methylation status for all markers. Performing multiple deamination reactions could not be done because of the limited amounts of biopsy DNA. We observed that fully methylated DNA deaminated and degraded at a slower rate than non-modified DNA. The methylation appeared to protect the DNA during bisulfite conversion. We modified the bisulfite protocol to introduce limited *in vitro* methylation at AluI and HaeIII sites on all DNAs prior to conversion. We expected the limited number of methylated cytosines in each CpG island to drive the complete deamination of unmethylated cytosines and help protect the DNA from degradation during bisulfite conversion.

In an effort to minimize the variability in conversion rates, we tested rapid deamination using ammonium salts and selected two deamination conditions (15 or 22 min at 70°C). The conditions were

selected based on their suitability for the deamination of *APC*, *RARB*, *GSTP1* (15 min) and *RASSF1* (22min). We wanted these markers included in the study to allow for the comparison of our results to published data. We selected additional markers that could be properly deaminated at 15 and 22 min. The conversion reactions were performed as described below. The length of bisulfite treatment is shown in Table 2 under Mix (15 or 22 min). SssI methylated CCL 119 DNA and AluI/HaeIII methylated lymphoblastoid DNA were also used as additional positive and negative controls respectively for the qPCR. Even though we added fully methylated and unmethylated lymphoblastoid DNAs as controls, they were not used to establish the bisulfite conversion conditions. Those were best selected based on the results obtained using several cancer cell lines. Control DNAs were added to each set of bisulfite conversions and carried forward through all amplifications to monitor the reproducibility of the experimental conditions.

Bisulfite treatment:

Ten nanograms of DNA were methylated sequentially *in vitro* using AluI and HaeIII methylases according to supplier's recommendation. Following the addition of 20 ng of fully methylated E. coli DNA (with SssI (CpG) and GpC methylases), the DNA was subjected to a saturated ammonium/sodium bisulfite treatment for 15 min or 22 min at 70°C. The ammonium bisulfite solution was prepared fresh immediately before use as follows: 0.67 grams of ammonium sulfite monohydrate (sigma-Aldrich) was dissolved in 5ml of 50% ammonium hydrogensulfite solution (Wako USA) by gently inverting the tube until fully dissolved. 2.2 grams of sodium metabisulfite was added to the ammonium solution and mixed by gentle inversion for 20 min. No heat was applied to increase the solubility of the sodium salt. The resulting solution is saturated with a significant amount of residual salt. The pH of each batch was checked before initiating the conversion reactions to verify that it was about 5.5 (using pH paper). Ten nanograms of methylated DNA were denatured in 25 μl total volume using 0.2M NaOH for 12 min at

37°C. 200 μl of the conversion solution were added, and the DNA was incubated at 70°C for 15 or 22 min in an Eppendorf thermocycler. The converted DNA was immediately diluted with 200 μl of H_2O and loaded on Amicon Ultra (50K) columns (Millipore). DNA clean-up was performed by spin filtration for 3 min at 10k. The DNA was washed once with 400 μl water, desulfonated on the column for 6 minutes in 0.3M NaOH (Fisher Scientific), washed 3X with 400 μl water and recovered in 35 μl H_2O. The DNA was stored frozen at -20°C until needed. We obtained sufficient chemical reagents from the same lots to perform all bisulfite reactions in order to avoid variations in deamination rates introduced by differences between lots of bisulfite salts.

Marker selection and assay optimization: Markers were selected based on an in-house marker screening protocol to identify CpG islands that are highly methylated in prostate cancer and unmethylated or methylated to a significantly lesser degree in BPH. Methylation-specific PCR assays were developed for over 2500 CpG islands with particular emphasis on genes mapping to 8p and 10q, 2 regions commonly deleted in prostate cancer. Additional markers were chosen based on biological function. The initial marker screening was performed on DNA converted using a standard sodium bisulfite conversion. Briefly, 1 microgram of PCA or BPH DNA was converted in 500 μl of sodium bisulfite solution for 8 cycles of 95°C for 5 min and 55°C for 110 min. Following purification, the DNA was resuspended in 100 μl of H_2O. Two microliters were used directly for MS-PCR.

Each marker was screened initially using 6 prostate cancer DNAs (from prostatectomies) and 6 BPH DNAs. Markers which were methylated in 2 or more prostate cancer DNAs and 2 or less BPH DNAs were chosen for screening on a larger panel of prostatectomy and BPH DNAs. The panel included 82 prostatectomy and 67 BPH samples. The deidentified tissue samples were provided by Dr. Daniel Murtagh. The marker screening resulted in over 100 markers that were preferentially methylated in cancer and suitable for the development of prostate cancer diagnostic tests.

For this biopsy marker panel, we included markers that were moderately to heavily methylated in prostate cancer. The 24 markers presented here were selected because they were the first markers we optimized that deaminated under the same conditions as *GSTP1*, *APC*, and *RASSF1*. *HOXD9* was slightly underdeaminated at 22 min. Some markers like *BAG4*, *ELGN3*, *FGF20* were overdeaminated at 15 min while other markers like *NODAL* and *HOXA7* were underdeaminated at 22 min. These markers would have required additional deamination reactions for which we did not have sufficient biopsy DNA. We did not attempt to optimize a qPCR assay for all markers identified during the screening and we did not use any clinical criteria for marker selection.

The markers selected for this study and the frequency of methylation based on the initial screening are: *ADCY4* (76%), *ARHGEF10* (62%), *CYBA* (90%), *CXCL14* (76%), *GFRA2* (74%), *GPX7* (70%), *GRASP* (57%), *HAPLN3* (59%), *HEMK1* (66%), *HOXB5* (68%), *HOXD9* (75%), *KIFC2* (54%), *KLK10* (60%), *LOXL2* (85%), *MOXD1* (50%), *NEUROG3* (70%), *RASSF5* (75%), *SLC16A5* (60%), and *SOCS3* (83%). Some of these markers were described by other researchers while this study was ongoing [52-57]. Please note that the frequency of methylation presented here is based on the sodium bisulfite conversion described above before we understood the need to optimize the length of bisulfite conversion for each marker. We can't predict how optimal screening conditions would affect the observed methylation frequencies. Furthermore, the qPCR assays may be derived from a different part of the CpG island than the original MS-PCR assays which may also affect the observed methylation frequency. The qPCR assays required 2 neutral primers (no CGs) that were separated by less than 300 bp. It was not always possible to find suitable primers from near the original MS-PCR assay used for screening. The CpG islands and chromosomal coordinates are listed in Table S1.

The primers, probes, length of bisulfite treatment and multiplex for each marker are listed in Table S2. All probes were labeled with FAM and quenched with BHQ1 (Biosearch Technologies, Petaluma, CA).

Unlabeled primers were obtained from Biosearch Technologies or Eurofins Genomics. The markers were grouped into 4 multiplexes (15a, 15b, 22a, 22b as listed in Table S2) and detected using a nested PCR strategy. The primers selected for the multiplex amplification were neutral (no CGs) or degenerate (at CGs) and were designed to amplify all templates regardless of methylation. Primers were also degenerate at positions of *in vitro* methylation because FFPE DNA is heavily cross-linked and the accessibility of all sites to enzymatic modification is unknown.

The bisulfite converted DNA was first preamplified with each primer multiplex to generate templates for the semi-quantitative MS-qPCR as follows: 5 μl of the bisulfite-treated DNA were subjected to 23 cycles of 95°C for 15 sec, 58°C for 45 sec, 72°C for 45 sec using the manufacturer's supplied buffer (adjusted to 2.5 mM $MgCl_2$) and dNTPs, 1 unit of Takara Taq polymerase HS (Clonetech), and 200 nM of each primer in the multiplex. The amplified DNA was diluted with 300 μl of H_2O. Four microliters of the diluted primary PCR reaction were used as input for the nested qPCR reaction.

To monitor the recovery of amplifiable DNA following bisulfite, the multiplex reactions were spiked with 2 control amplicons. We couldn't check for the recovery of each individual marker using unmethylated primers to amplify the unmethylated templates because of the variable rates of degradation of methylated and unmethylated templates. Instead, we chose 2 control markers that are recovered reliably under the chosen bisulfite treatment conditions. The amplification of the controls indicates the recovery of amplifiable DNA following bisulfite conversion. For the 22 min bisulfite reaction, we used the *ADCY4* amplicon which was already in Mix 22a. For the 15 min reactions, we used primers for *NT5E* in Mix 15a. The primers for the DNA quality control PCR reactions are listed in Table S2. DNA control reactions were performed on an Eppendorf Mastercycler using 4 μl of the diluted primary multiplex PCR for 35 cycles of 95°C for 20 sec, 60°C for 20 sec, 72°C for 45 sec using the manufacturer's supplied buffer

and dNTPs with 2.5 mM $MgCl_2$, 0.5 unit of Takara Taq polymerase HS, and 1.0 μM of forward and reverse primers.

Each qPCR reaction was performed in duplicate for 40 cycles using the manufacturer's supplied buffer and dNTPs with 2.5 mM $MgCl_2$, 0.5 unit of Takara Taq polymerase HS, 0.66 μM forward primer (same orientation as the probe), 1.3 μM reverse primer and 0.5 to 1 μM of the probe on an Illumina Eco qPCR Real-Time PCR system (Illumina, San Diego, CA). The reaction conditions for each assay are listed in Table S2.

The bisulfite conditions selected for each marker yielded no detectable amplification from negative controls (lymphoblastoid DNA and at least 1 of the cancer cell line DNAs) for DNA concentrations ranging from 0.625 ng to 20 ng. All MS-qPCR assays reliably detected their respective markers from the 2.5 ng bisulfite reaction from at least 1 cancer cell line or PCA tumor DNA and the *in vitro* SssI-methylated control DNA. The reference DNA concentration range for the deamination conditions used in this study is 2.5 ng to 20 ng. For some markers like APC, the lower limit of detection was 0.625 ng. The quantitative PCR assays were further optimized using serial dilutions of the 10 ng bisulfite reaction from a positive cancer cell line usually PC3 or DU145.

__Data collection__: The data was tabulated using the Eco Study application provided with the Illumina ECO Real-Time PCR system. A cutoff of 35 for the Cq was used as the upper limit for a positive reaction for all markers which is supported by the data generated during marker optimization. The majority of samples never showed any amplification (no increase in fluorescence) which was recorded as 0 (no detectable methylation). The Cq for the positive controls was between 20 and 25 cycles depending on the marker and represented the highest concentration achievable for each marker under the analytical conditions used. A higher Cq represents a lower number of methylated copies in the sample. In general, every 1 cycle decrease in Cq reflects a doubling of the number of methylated templates as determined using

serial dilution of the positive controls during assay optimization. A cutoff of 35 yielded a good balance between minimizing false positives and false negatives and was acceptable for a marker survey study.

The data was further transformed by subtracting the Cq values from 35 (except for the 0 data points) to generate an increasing range of Cqs from 0 (no amplification) to 15 (highest level of amplification). The data was used directly for statistical analysis with no further manipulations.

Table S1. Chromosomal location of CpG islands

Gene Symbol	Gene ID	Length	Chr	Begin	End	mRNA ID	Position relative to 1st UTR of
**ADCY4**	196883	1050	14	23,873,401	23,874,451	NM_139247.2	-747 to 303
**APC**	324	550	5	112,101,037	112,101,587	NM_000038.3	-446 to 104
**ARHGEF10**	9639	1300	8	1,771,401	1,772,701	NM_014629.2	-748 to 552
**CXCL14**	9547	850	5	134,942,137	134,942,987	NM_004887.3	-119 to 731
**CYBA**	1535	800	16	88,717,034	88,717,834	NM_000101.2	-377 to 423
**GFRA2**	2675	1800	8	21,702,091	21,703,891	NM_001495.4	-1599 to 201
**GPX7**	2882	1050	1	52,779,716	52,780,766	NM_015696.2	-349 to 701
**GRASP**	160622	1550	12	52,400,345	52,401,895	NM_181711.2	-403 to 1147
**GSTP1**	2950	800	11	67,107,513	67,108,313	NM_000852.2	-349 to 451
**HAPLN3**	145864	800	15	89,438,124	89,438,924	NM_178232.2	-154 to 646
**HEMK1**	51409	800	3	50,579,767	50,580,567	NM_016173.1	-3599 to -2799
**HOXB5**	3215	550	17	46,670,499	46,671,049	NM_002147.3	54 to 604
**HOXD9**	3235	2050	2	176,986,483	176,988,533	NM_014213.3	-930 to 1120
**KIFC2**	90990	1300	8	145,668,447	145,669,747	NM_145754.2	5901 to 7201
**KLK10**	5655	1300	19	51,521,683	51,522,983	NM_145888.2	-29 to 1271
**LOXL2**	4017	1300	8	23,316,624	23,317,924	NM_002318.1	-349 to 951
**MOXD1**	26002	1050	6	132,763,665	132,764,715	NM_001031699.1	-390 to 660
**NEUROG3**	50674	1300	10	71,002,177	71,003,477	NM_020999.1	-349 to 951
**PTGS2**	5743	800	1	186,649,009	186,649,809	NM_000963.2	-250 to 550
**RARB**	5915	1200	3	25,468,654	25,469,854	NM_016152.3	-1100 to 100
**RASSF1**	11186	1050	3	50,352,651	50,353,701	NM_007182.4, and others	-330 to 720
**RASSF5**	83593	1550	1	203,068,425	203,069,975	NM_031437.1, and others	-849 to 701
**SLC16A5**	9121	800	17	73,083,749	73,084,549	NM_004695.2	-306 to 494
**SOCS3**	9021	2050	17	76,354,749	76,356,799	NM_003955.3	-641 to 1409

Table S1 shows the list of the CpG islands and associated genes and chromosomes. The coordinates of the CpG islands on the chromosome are listed for build 38. The position relative to the first untranslated region (UTR) is ”–“ if it is 5’ to the UTR or “+” if it extends into the UTR based on the mRNA ID provided.

Table S2. qPCR Assays Primers and Conditions

Gene	Primers and Probes	Mix	qPCR conditions
ADCY4	BNF: TAGGAGGGTAGGATTTGGGGTTG<br>BNR: CTATCRCCCRAAACTCTAAACCAA<br>F: GTAGCGGAGTGGGTTAGGTTC R: TCGCCCGAAACTCTAAACCAA<br>Probe: TTTGATGGCGGAGTTACGTTCGTCG	22a	95°C for 15 sec,<br>68°C for 20 sec,<br>65°C for 20 sec
ADCY4	Control ADCY4 Primers: BNF1: GTAGYGGAGTGGGTTAGGTT<br>BNR1: TCRCCCRAAACTCTAAACCAA		See Materials and Methods
APC2	BNF: GYGGAGAGAGAAGTAGYTGTGTAA<br>BNR: ACAACACCTCCATTCTATCTCCAA<br>F: TCGTTGGATGCGGATTAGG R: CCTAACGAACTACACCAATACAAC<br>Probe: CGTCGGGAGTTCGTCGATTG	15a	95°C for 15 sec,<br>68°C for 20sec,<br>62°C for 20 sec
ARHGEF10	BNF: GGGGTAGGAGATGATTYGGG<br>BNR: ACCTACTCCACCCACCATACTATC<br>F: GYTACGTGGYTTCGAAGG R: CGTTCCGAACGCCCATTARC<br>Probe: CGTTGTCGAGAAATCGGTAGGTTTTGCG	15b	95°C for 15 sec,<br>69°C for 20 sec,<br>65°C for 20 sec
CXCL14	BNF: GGGATTTTAGGATGTTTAGAAATTG<br>BNR: CTCCCCATATCCCTACTCCCAC<br>F: CGCGGTGTATAGCGTTAG R: GCCGCGCCCCTCCGATCAAC<br>Probe: TAGCGCGGYCGTTAGGAGT	22a	95°C for 15 sec,<br>67°C for 20 sec,<br>62°C for 20 sec
CYBA	BNF: GAGGTTTYGGTTGGGGTTTTGG<br>BNR: AAACAACCCTACACCCTACAAATAC<br>F: CGGACGTTAGCGTTTGTTC R: CGACAATACGCGCCTAACAAT<br>Probe: TTATGGCGATACGAATTCGGTTGGGA	22b	95°C for 15 sec,<br>69°C for 20 sec,<br>65°C for 20 sec
GFRA2	BNF: GGTTAGGGGAGGAGGAATTTTTT<br>BNR: CCTTCCCCAACCRATCTCCCCTC<br>F:GGAGTCGGGAGTTGTTTC R: ACCACCTCCTCGAAARCCGAC<br>Probe: AGTCGGTTGCGGAGTTTCGG	15a	95°C for 15 sec,<br>68°C for 20 sec,<br>63°C for 20 sec
GPX7	BNF: TTATYGTTTGYGGAGGGAAYGAGAA<br>BNR: CTTCTCCAACRACACCAATTTACCC<br>F: GTCGAGCGGCGTTAGAGT R: ATCCTACTCCTACTACGCGCAA<br>Probe: CCGCCACCATAACTTATTCCGAAA	15b	95°C for 15 sec,<br>67°C for 20 sec,<br>62°C for 20 sec
GRASP	BNF: TTAGGAAGYTGTAGTAGAAGGAGG<br>BNR: ACRAAACAAAATACCCCCRAACAC<br>F: TATTTCGGATTTCGTCGTTCGGA<br>R: AACACGACGAACGCGCGATAC<br>Probe: TTCGATTCGGAAGTCGCGTTCGTC	22b	95°C for 15 sec,<br>69°C for 20 sec,<br>65°C for 20 sec
GSTP1	BNF: GGATTTGGGAAAGAGGGAAAGGTTT<br>BNR: AAARCTCTAAACCCCATCCCC<br>F: TAGYTGCGCGGCGATTTCGG R: CGAAAACTACGACGACGAAACTC<br>Probe: AAATCCCGCGAAMTCCCGCCGRC	15b	95°C for 15 sec,<br>68°C for 20 sec,<br>62°C for 20 sec

HAPLN3	BNF: GGATTTTGTTYGGGAGGTGTGG<br>BNR: TTTCTACTACCRCTTCCTTTCTAC<br>F: GCGGTAAGGGAGGAATTCGG R: TTAAACCGAAACARCTCGCGRC<br>Probe: TTTTCGACGGTTAGAGYTTTCGGG	22b	95°C for 15 sec,<br>68°C for 20 sec,<br>62°C for 20 sec
HEMK1	BNF: GGTGGTAGTGATTGGTAGGAGG<br>BNR: ACCCTACCCACCCTAACTCAA<br>F: GTTGCGGGTTCGATTCGAGATT R: CCCACCCTARCTCAARCCGA<br>Probe: TGCGTATTAGTCGTATTCGCGAGCG	22a	95°C for 15 sec,<br>68°C for 20 sec,<br>62°C for 20 sec
HEMK1rc	BNF: GTGTAGGGAGGTAAGGYGTTGTAG<br>BNR: AAATCCRCCCTCCCTACAAACCC<br>F: GTCGTGGGTTTCGGYTTT R: TARCCAARCCCGCTACTAC<br>Probe: CGTTCGCGGATGCGGTTGGTG	22a	95°C for 15 sec,<br>68°C for 20 sec,<br>63°C for 20 sec
HOXB5	BNF: GGTGGYTATGGGTTTTGGTTGYGTT<br>BNR: ACCTCAACRTCAACCRCTCCTCRAC<br>F: CGAGGACGCGTTGGTTTC R: ACGACGCCAAACCCTCTACTTC<br>Probe: ATTTCGGTGAAATTGGCGTTGGAGYT	22b	95°C for 15 sec,<br>69°C for 20 sec,<br>65°C for 20 sec
HOXD9	BNF: GATTAATGGTGGAGGTTGTAGTTTG<br>BNR: ARCCATCAACCACACCTACAARC<br>F: TTGCGAATTAGTCGGTGGTTC R: CTCGTCGCCCTCATARCC<br>Probe: TACACTATCCGCCGCCGAACARCTC	22b	95°C for 15 sec,<br>69°C for 20 sec,<br>65°C for 20 sec
KIFC2-1	BNF: GTGGAGATTTGYGGGTAAGAGG<br>BNR: CCTACRAAAARCCCAAACCATAAACC<br>F: GTATCGCGGTGGTGTTTG R: CGCTCGCTACTAACGCTAA<br>Probe: AGCGTCGGTTGTAGTAGTCGCGTG	22a	95°C for 15 sec,<br>68°C for 20 sec,<br>63°C for 20 sec
KIFC2-2	BNF: GTGTTTAGAGGGGYGAGYGAGTT<br>BNR: CCCTACACCTAACCAACCCTTC<br>F: AGCGCGCGGTGGAGACGT<br>R: GCCACCGCCATAAACCAACGCA<br>Probe: TCGCGCGTAGCGTTAGCGTGATT	22a	95°C for 15 sec,<br>68°C for 20 sec,<br>63°C for 20 sec
KLK10	BNF: GAGTTAGAAGAAGGGYTTAGYTGA<br>BNR: ARCTCCRCACCTCCACCTCTCC<br>F: GGYCGTGTTTCGGAGCGTT R: AACGCCCGAACTCTAACGAA<br>Probe: TGCGCGGGGYTTGGTGACGG	15b	95°C for 15 sec,<br>69°C for 20sec,<br>65°C for 20 sec
LOXL2	BNF: AGTGTAGYGTGGTTTTAGGGATAG<br>BNR: CCTCTACTCCCRCAAAACTCAC<br>F: GGTCGGGAGATAGTGATTGTTT R: GCTCCCAACAACCTTTCGA<br>Probe: TGCGTTCGGGAGYTGTAGCGTT	15a	95°C for 15 sec,<br>68°C for 20 sec,<br>63°C for 20 sec
MOXD1	BNF: TTTGGAGGTAGGGYYGTTYGTGG<br>BNR: CTACTCCTACTATAAAAACTACTCC<br>F: GTCGGCGGACGTTATGGYT R: CCGCCTCCAAATACGCACTAC<br>Probe: TGGGCGAGAAGTCGAAGTTTACGTA	15b	95°C for 15 sec,<br>69°C for 20 sec,<br>62°C for 20 sec
MOXD1rc	BNF: GGAGGGTAAGTATTGGTTGGGTT<br>BNR: CACCTACRCCCTCTCTAAAATCC<br>F: GCTATGGCGTTCGTCGATA R: CCTACGCCCTCTCTAAAATCCGA<br>Probe: TCGGAGTTCGTTCGGYCGAGTTCG	15a	95°C for 15 sec,<br>68°C for 20 sec,<br>63°C for 20 sec

NEUROG3	BNF: GGYTTAAGAGYGAGTTGGTATTGAG<br>BNR: AACCRCCTAAACTACCCARCTCC<br>F: AGACGACGCGAAGYTTATTAAGATC<br>R: TCCAACGCGTACAARCTATAATCC<br>Probe: TTGGGCGTTGATTTAAACGTTGCGT	15a	95°C for 15 sec,<br>68°C for 20 sec,<br>62°C for 20 sec
NEUROG3 rc	BNF: TAGTTTGGGAGATTGGGGAGTAG<br>BNR: CAATCRAATACACAACCTCAACTC<br>F: GCGTTTGAGTTAGCGTTTAGA R: ACGCCCTACGCGATATCCTAC<br>Probe: TTTGGTGAGYTTCGCGTCGTTTG	15a	95°C for 15 sec,<br>68°C for 20 sec,<br>63°C for 20 sec
PTGS2	BNF: GGAGTATTGGGATAGATTTAGGAG<br>BNR: AAACTCCTCAACAACRCCTCCTTCA<br>F: GTGCGTGGATTCGGAGTT R: CCAAACGCCCTCAAACAACA<br>Probe: CGCGGGCGAGTATCGTAGCG	15a	95°C for 15 sec,<br>68°C for 20 sec,<br>63°C for 20 sec
RARB	BNF: GTAAAGGGAGAGAAGTTGGTGTTT<br>BNR: ACCCTACCCRAACTACTAACCTTC<br>F: CGTGAGTTAGGAGTAGCGTT R:<br>CTAACATCACCAACTCCCAAAATTC<br>Probe: AACGTCGGTTTGTGCGTTCGTTG	15a	95°C for 15 sec,<br>68°C for 20 sec,<br>63°C for 20 sec
RASSF1	BNF: GGGTGTTAGYTTTYGTAGYTTAATGAG<br>BNR: CCACAATCCCTACACCCAAATTTCC<br>F: TCGGTTGGGYTCGTGTTTC R: ATCGAARCCCGCCCTATARCC<br>Probe: CCGRCCCGCGCTTACTAACGC	22a	95°C for 15 sec,<br>68°C for 20 sec,<br>63°C for 20 sec
RASSF5-1	BNF: TAGGGTTGYGAAGGAAGGGGAA<br>BNR: ATCTAAATAAARCCCCTCCRTCTAAAC<br>F: TAGGGTTGCGAAGGAAGG R: CGCGCTTTATCCCGTAAATAACC<br>Probe: ATTAGAGCGAAAGTCGTACGCGGA	22a	95°C for 15 sec,<br>68°C for 20 sec,<br>62°C for 20 sec
RASSF5-2	BNF: TTTYGGYTTGGTTTGTAGTAGAGATTG<br>BNR: CCCTCCCCTACCTTCACACTTAC<br>F: CGGAGTATTTTCGAGTAGTCG R: CCTCTCGTCCGCACAAATCA<br>Probe: TTTCGGCGGAGCGAGGCGAG	15a	95°C for 15 sec,<br>68°C for 20 sec,<br>62°C for 20 sec
SLC16A5	BNF: GGAGGTTGGAAGGTGGGTTTT<br>BNR: CTTTTCTAAATCCTCACCRCCACT<br>F: CGGCGTTTGGYTTAGTTTC R: AACGCGTCCGAAACAATAAARCCC<br>Probe: TTCGTTAGGYTCGTGCGAGCGGG	22b	95°C for 15 sec,<br>69°C for 20 sec,<br>65°C for 20 sec
SOCS3	BNF: GGTATGTAGTGGTGTATTAGTTTG<br>BNR: CCTAAACACCAACCTACRCCTCA<br>F: GACGTTGAGCGTGAAGAAGTG R: AACCCGCCGACACCTTTC<br>Probe: CGTTGGTTCGAGYTGTCGCGGATTA	22b	95°C for 15 sec,<br>69°C for 20 sec,<br>65°C for 20 sec
NT5E	BNF: GGYGATTAGTATTAGGGTATTATTTG<br>BNR: AACTTCTATACATCCAACTAACACAC<br>BNF1: GTATTAGGGTATTATTTGGTTTATYGTG<br>BNR1: TCTCACTTTATCCCCACATCTCTCC	15b	See Materials and Methods

Table S2 shows the primers and conditions used for the MS-qPCR assays. For each gene, 2 sets of primers are provided. The BNF and BNR primers were the neutral primers used for the multiplex amplification as described in Materials and Methods. The F, R and probe primers were used for the qPCR assays. All probes were dual labelled with FAM and BHQ1. All primers were made degenerate at positions of *in vitro* methylation. The conditions listed were optimized for the Illumina Eco Real Time PCR system. The Mix number listed for each set of neutral primers defines the multiplex that the primer pair was used in. 22 and 15 represent the length in minutes of the bisulfite treatment used to analyze the corresponding marker.
